# A mobile phone-based program to promote healthy behaviors among adults with prediabetes: study protocol for a pilot randomized controlled trial

**DOI:** 10.1186/s40814-018-0246-z

**Published:** 2018-02-13

**Authors:** Dina H. Griauzde, Jeffrey T. Kullgren, Brad Liestenfeltz, Caroline Richardson, Michele Heisler

**Affiliations:** 1 Robert Wood Johnson Foundation Clinical Scholars Program, University of Michigan, Ann Arbor, MI USA; 2 VA Center for Clinical Management Research, VA Ann Arbor Healthcare System, 2800 Plymouth Road, Building 14, Room G100-36, Ann Arbor, MI 48109-2800 USA; 3 University of Michigan Medical School, Ann Arbor, MI USA; 4 University of Michigan Institute for Healthcare Policy and Innovation, Ann Arbor, MI USA; 5 University of Michigan School of Nursing, Ann Arbor, MI USA

**Keywords:** Prediabetes, Type 2 diabetes mellitus, Prevention, Autonomous motivation, Behavior change

## Abstract

**Background:**

Rates of participation in Diabetes Prevention Programs (DPPs) are low. This may be due, in part, to low levels of autonomous motivation (i.e., motivation that arises from internal sources and sustains healthy behaviors over time) to prevent type 2 diabetes (T2DM) among many individuals with prediabetes. Mobile health (mHealth) technologies that incorporate principles from the Self-Determination Theory offer an effective and scalable approach to increase autonomous motivation levels. One promising mobile phone-based application is JOOL Health, which aims to help users connect certain health behaviors (e.g., sleep and diet) with personal values in specific life domains (e.g., family and work). The first aim of this study is to estimate whether JOOL Health can increase autonomous motivation to prevent T2DM among individuals with prediabetes who declined DPP participation. The second aim of this pilot study is to examine the intervention’s feasibility and acceptability.

**Methods:**

This is a 12-week, three-arm pilot randomized controlled trial. We will recruit 105 individuals with prediabetes who did not engage in a DPP despite invitation from their health plan to participate in face-to-face or web-based programs at no out-of-pocket-cost. Participants will be randomized to one of three study arms: (1) a group that receives information on prediabetes, evidence-based strategies to decrease progression to T2DM, and a list of resources for mHealth tools for monitoring diet, physical activity, and weight (comparison group); (2) a group that receives the JOOL Health application; and (3) a group that receives the JOOL Health application as well as a Fitbit activity tracker and wireless-enabled scale. Our primary outcome is change in autonomous motivation to prevent T2DM (measured using the Treatment Self-Regulation Questionnaire). We will also collect data related to the intervention’s feasibility (recruitment and retention rates) and acceptability (adherence and qualitative experience) as well as changes in psychosocial outcomes, hemoglobin A1c, and weight.

**Discussion:**

To our knowledge, this is the first study that aims to promote positive health behaviors among individuals with prediabetes who previously declined to participate in a DPP. Our results will inform a larger trial to test the effect of JOOL Health on clinically relevant outcomes, including weight loss, physical activity, and DPP engagement.

**Trial registration:**

NCT03025607. Registered February 2017.

**Electronic supplementary material:**

The online version of this article (10.1186/s40814-018-0246-z) contains supplementary material, which is available to authorized users.

## Background

Within the USA, type 2 diabetes mellitus (T2DM) and its precursor, prediabetes, are growing public health concerns. Approximately 84 million adults have prediabetes [1], and it is estimated that one-third of the adult population will have T2DM by 2050 [2]. Diabetes Prevention Programs (DPPs) can promote modest weight loss [3–8] to help individuals with prediabetes avoid progression to T2DM [9–11], and these programs are available in communities throughout the USA [4, 12].

Despite nationwide availability of the DPP [4] and public health initiatives to identify and treat individuals with prediabetes [13, 14], rates of DPP engagement are extremely low [15]. Barriers to DPP participation include logistical factors (e.g., transportation, time, cost) [16] and behavioral factors (e.g., motivation, risk perception) [17–19]. Although web-based DPPs [20, 21] and growing insurance coverage for program participation [22–24] can reduce logistical barriers, these strategies alone have been inadequate to increase rates of DPP engagement. In September 2015, for example, a single University’s self-funded health insurers began to offer face-to-face and online DPP options to healthcare plan members (i.e., employees, retirees, and students of the University or their dependents) with prediabetes at no out-of-pocket-cost, yet only 6% of invitees enrolled in a DPP within 6 months. Prior work demonstrates an association between greater levels of autonomous motivation and increased engagement in healthy behaviors among this university’s employees with prediabetes [25]. This finding, in combination with low levels of program engagement, suggests that behavioral barriers may more strongly influence rates of DPP participation than logistical ones.

New strategies are needed to motivate individuals with prediabetes to engage in healthy behaviors to prevent T2DM. Mobile health (mHealth) technologies that incorporate principles from behavioral change theories such as Self-Determination Theory (SDT) may be an effective and highly scalable approach [26]. SDT provides a conceptual framework for understanding human behavior across a continuum of motivation ranging from controlled motivation to autonomous motivation [27]. In contrast to controlled motivation, which originates from a sense of external obligation or pressure, goals and behaviors are “autonomous” when they align with personal interests and values [28]. Greater levels of autonomous motivation correlate positively with dietary adherence [29], weight loss [30, 31], physical activity [32, 33], and DPP participation [34]. Importantly, autonomous behaviors are sustained over time [35, 36].

JOOL Health is a mobile phone-based health application that aims to increase autonomous motivation by helping users connect certain health behaviors (e.g., sleep, diet, physical activity) with personal values in specific life domains (e.g., family and work) [37]. JOOL Health integrates user-entered information with contextual data (e.g., weather and day of the week) and then delivers tailored messages to help individuals gain awareness of and control over the factors that contribute to their well-being and ability to engage in self-care behaviors. In this way, JOOL Health aims to cultivate autonomous sources of motivation to initiate and maintain healthy behaviors. Importantly, the JOOL Health application has been tested among heterogeneous populations including healthy volunteers and individuals with chronic diseases. These unpublished data demonstrate high user engagement and statistically significant increases in self-reported health behaviors.

We propose a pilot study to estimate the ability of JOOL Health—used alone and also in conjunction with Fitbit devices (i.e., activity tracker and wireless internet-enabled scale)—to increase autonomous motivation to prevent T2DM among individuals with prediabetes who declined participation in a formal DPP (i.e., DPP non-participants). Given that JOOL Health draws on SDT to inspire behavioral change, we hypothesize that autonomous motivation to prevent T2DM will increase to a greater degree among individuals who use JOOL Health compared to individuals who only receive written educational materials about T2DM prevention. Because Fitbit devices can also enhance user motivation by fostering autonomy, competence, and relatedness (through sharing of fitness data) [38]—key SDT tenets of psychological need—we also hypothesize that autonomous motivation to prevent T2DM will increase to a greater degree among individuals who use JOOL Health in conjunction with Fitbit devices compared to individuals who use the JOOL Health application alone. Further, we aim to examine the feasibility of recruiting DPP non-participants and the acceptability (adherence and qualitative experience as reported during semi-structured interviews) of the intervention. We hypothesize that recruiting DPP non-participants may have unique challenges, as these individuals have already declined to participate in one lifestyle intervention. However, as compared to the DPP, this is a low-intensity intervention, which may appeal to individuals seeking a less time-consuming program, and we believe that study participants will find the intervention acceptable. Taken together, these data will inform the design and implementation of a larger trial to test the effect of JOOL Health on clinically relevant outcomes, including weight loss, physical activity, and DPP engagement.

## Methods

**Fig. 1:**
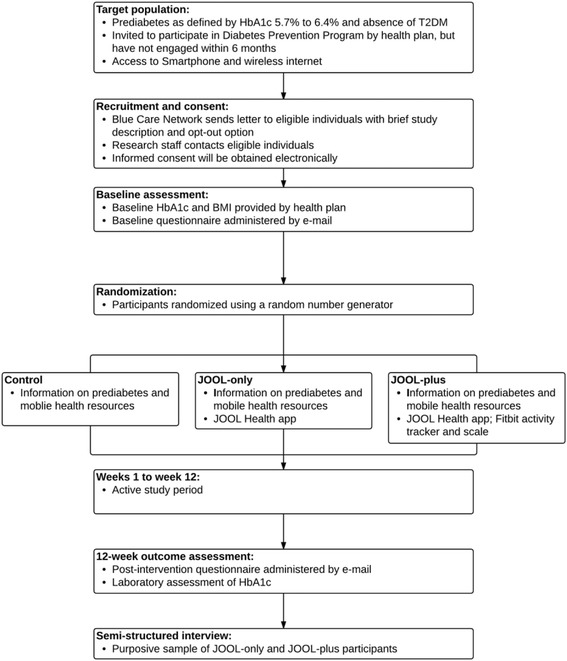
Study flow diagram

This study was approved by the University of Michigan Institutional Review Board. The protocol was designed according to the Standard Protocol Items: Recommendations for Interventional Trials 2013 (SPIRIT). A SPIRIT figure (Fig. 1) and SPIRIT checklist (Additional file 1) are provided. The study is funded by the Blue Cross Blue Shield of Michigan Foundation, and it is registered on ClinicalTrials.gov (NCT03025607).

### Study design

This study is a 12-week pilot randomized controlled trial. Participants will be randomized to one of three arms: (1) a group that receives information on prediabetes and evidence-based ways to decrease progression to T2DM as well as a list of resources for mHealth tools for monitoring diet, physical activity, and weight (comparison); (2) a group that receives the same information as group 1 and also the JOOL Health mobile phone application (JOOL-only); and (3) a group that receives the same information as group 1 and also the JOOL Health mobile phone application and Fitbit devices (i.e., activity tracker and wireless internet-enabled scale) whose results can be uploaded to JOOL Health (JOOL-plus). We will use a mixed methods approach with a sequential explanatory design, which is to say that quantitative data and qualitative data will be collected in two consecutive phases within the study [39]. Specifically, in the first phase, we will collect and analyze the quantitative data (e.g., surveys and weight). In the second phase, we will collect and analyze qualitative, semi-structured interview data from a purposive sample of 20 participants with differing levels of engagement and success with the intervention. The rationale for this approach is that the qualitative data will help to clarify these findings by exploring participants’ experiences and perspectives in more depth [40]. Additionally, through qualitative interviews, we will explore the acceptability of the JOOL Health application among JOOL-only participants and the acceptability of the JOOL Health application and Fitbit devices among the JOOL-plus participants.

### Study setting

The intervention will be delivered remotely. Individuals in each arm of the study will receive study materials by postal mail and/or e-mail. A study team member will be available by telephone to help participants troubleshoot technical issues that may arise with the study devices and to address study-related questions or concerns.

### Study participants

Inclusion criteria are (1) non-participation in a formal DPP at least 6 months after invitation to participate by the University’s self-funded insurers, Premier Care or Grad Care; (2) prediabetes based on the American Diabetes Association criteria of hemoglobin A1c (HbA1c) 5.7 to 6.4%; (3) access to a personal smartphone; and (4) access to home wireless internet. We will exclude women who are pregnant or intend to become pregnant during the intervention period. All eligible study participants are employees, retirees, and students of this university or their dependents.

### Recruitment

A member of the study team will contact by telephone a random subset of Premier Care and Grad Care DPP non-participants. Individuals interested in study participation will then be screened to ensure they meet study eligibility criteria, and informed consent will be obtained electronically using the RedCap survey platform [41].

### Allocation

Individuals who meet study inclusion criteria and consent to study participation will be randomized using a web-based tool, the University of Michigan computerized randomization system (TATUM—Treatment Assignment Tool-UM), which allows for blinded treatment allocation. We will use stratified randomization with variable block lengths to ensure a balance of age and gender between groups. Recruitment and randomization will be ongoing until we have 35 individuals in each arm of the study.

### Intervention

All participants (*N* = 105) will receive information regarding prediabetes, evidence-based T2DM prevention strategies, and mHealth resources to help monitor food intake and composition, weight, and physical activity (e.g., MyFitnessPal). All participants will be encouraged to engage in self-monitoring and will be provided information on the benefits of daily weighing and monitoring of food and physical activity.

Participants in the JOOL-only and JOOL-plus arms will receive instruction by e-mail on how to use their respective mHealth applications. Participants in both intervention arms will be asked to use JOOL Health on a daily basis. JOOL Health will deliver tailored messages to individuals based on the daily information they enter in the following behavioral health domains: sleep, presence, activity, creativity, and eating. Additionally, the tailored messaging will be informed by contextual factors (e.g., local weather and day of the week). Participants in the JOOL-plus arm will also be asked to perform daily weights using a Fitbit wireless internet-enabled scale and to wear a Fitbit physical activity tracker. These devices will interface with the JOOL Health platform, and the data will inform JOOL-delivered tailored messages.

### Outcome measures

#### Primary outcome measures

The primary outcome will be change in autonomous motivation to prevent T2DM. Autonomous motivation will be measured using the seven-item, validated Treatment Self-Regulation Questionnaire at baseline and 12-week follow-up [42].

#### Secondary outcome measures

The feasibility of recruitment will be determined by calculating the intervention uptake rate, defined as the number of participants recruited to the intervention divided by the total number of eligible participants. Because we may not be able to reach all eligible participants via telephone (e.g., disconnected phone line), we will also calculate the rate of intervention uptake among only those for whom we are able to contact. Reasons for non-participation in this study will be recorded.

Among JOOL-only and JOOL-plus participants, we will calculate rates of adherence to the JOOL Health application, defined as the number of app-usage days (defined as the number of days that users entered data into JOOL Health) divided by the total number of days during the intervention period. Among JOOL-plus participants, we will calculate rates of participant adherence to the Fitbit activity tracker and scale, defined as the number of total days that each of these devices were used divided by the total number of days during the intervention period.

Participant retention will be determined by calculating the completion rate for the 12-week survey.

The acceptability of the intervention will be determined through semi-structured interviews conducted via telephone. Following the intervention period, we will conduct semi-structured interviews with JOOL-only and JOOL-plus participants. For purposive sampling, we will interview individuals with differing levels of engagement with the intervention and change in autonomous motivation using cut-points for high and low levels of these determined following quantitative data analysis. We will conduct at least 20 interviews divided equally between JOOL-only and JOOL-plus participants; additional interviews will be conducted if we do not reach thematic saturation after 20 interviews [43, 44]. During the interviews, we will explore the following: reasons participants chose to engage in this study; participants’ experience with the JOOL Health application; and participants’ experiences with the Fitbit activity tracker and scale, if applicable. In this way, we will gain insight into the particular intervention characteristics (or combination of characteristics) that may have facilitated or hindered the participants’ ability to achieve the primary outcome. We will also solicit participants’ ideas regarding additional behavioral change supports and their recommendations on ways to improve the intervention.

We will evaluate change in HbA1c; baseline HbA1c will be abstracted from the electronic medical record and participants will be asked to have a follow-up HbA1c drawn after the 12-week intervention period. Additional exploratory outcomes will include change in the following self-reported measures: weight (kg) and/or BMI (kg/m^2^); change in overall level of motivation to prevent T2DM [34]; purpose in life [45, 46]; perceived competence to prevent type 2 diabetes [47]; social support [48]; eating behavior [49]; self-reported physical activity [50]; patient activation [51]; and willingness to participate in a Diabetes Prevention Program. We will collect these exploratory data at baseline and 12-weeks using survey instruments, which will be administered using RedCap, a secure web application [52]. At baseline, we will also collect sociodemographic characteristics including age, gender, race, ethnicity, education, and income. Among JOOL Health users, we will also evaluate changes in charted daily health behaviors (e.g., sleep, eating, physical activity) from baseline (first 2 weeks of the intervention) to 12-weeks (last 2 weeks of the intervention). Among participants in the JOOL-plus arm, we will also assess objective change in physical activity minutes and body weight as measured with the Fitbit activity tracker and scale from baseline (first 2 weeks of the intervention) to 12-weeks (last 2 weeks of the intervention). Because the Fitbit devices interface with the JOOL Health platform, Fitbit data will be stored within the JOOL Health application. At the end of the study period, the JOOL Health team will provide our study team with a comma-separated values (CSV) file containing raw data from the JOOL app and Fitbit devices.

### Sample size

Based on prior studies of autonomous motivation among University of Michigan employees [34], we anticipate that the baseline level of autonomous motivation to prevent T2DM among Premier Care or Grad Care members who decline DPP participation will be 5.7 (measured on a 1 to 7 scale with 1 being the lowest and 7 being the highest). During the 12-week intervention period, we anticipate that autonomous motivation will increase by 0.6 points in the JOOL-only arm and by 0.8 points in the JOOL-plus arm. Assuming a standard deviation of 1.0 for change in autonomous motivation in both arms, 29 participants in each arm will provide 80% power to detect these changes in autonomous motivation in the intervention arms compared to the comparison arm. Prior research demonstrates that a 0.5-point increase in autonomous motivation can lead to greater weight loss and increased physical activity compared to individuals who did not achieve this increase in autonomous motivation [36]. To account for the possibility that some participants may be lost to follow-up during our 12-week intervention, we have conservatively inflated our sample size by 20% to enroll 35 participants in each arm.

### Data analysis

For all continuous outcomes, we will calculate changes from baseline to 12 weeks. For each continuous outcome, we will then conduct two sample *t* tests to determine whether the mean change for that outcome was statistically significantly different for each intervention arm relative to the comparison arm. For all categorical outcomes, we will use chi-square or Fisher’s exact tests to between-arm changes from baseline to 12 weeks. While we will be conducting multiple tests for statistical significance, we will not adjust our threshold for statistical significance (e.g., using a Bonferroni correction) for multiple comparisons so as to avoid making a type II error in this pilot study. We will conduct all analyses using Stata 14.

Semi-structured interviews will be audio recorded and subsequently transcribed verbatim. Interviews will then be imported into qualitative analysis software. Two investigators will independently read and code transcribed interviews. Interviews will then be coded jointly using consensus conferences. Interviews will be analyzed using directed content analysis [53].

Consistent with a mixed-methods sequential explanatory design [40], we will integrate (i.e., connect) the quantitative and qualitative findings in the final stage of data analysis. In this way, we will interpret our quantitative data in the context of qualitative participant experience.

## Discussion

Evidence-based Diabetes Prevention Programs are available in communities across the USA and can effectively promote weight loss among individuals with prediabetes [12]. However, the DPP alone may be insufficient to improve population health, as low rates of participation limit the program’s reach. Prior studies have described determinants of participation [17] and non-participation [16, 54, 55] in lifestyle-change programs, as well as barriers to adherence among those who initially engage in these programs [19]. However, it is unknown whether interventions to increase levels of autonomous motivation—a key driver of lifestyle change among individuals with prediabetes [25]—can promote healthy behaviors and greater rates of DPP engagement among individuals with prediabetes.

In this pilot study, we aim to test whether JOOL Health can increase autonomous motivation among individuals with prediabetes when used alone and also in conjunction with other mHealth tools. Because changes in individuals’ levels of autonomous motivation occur early along the path to behavioral change, we anticipate a large effect size from the intervention and will therefore have the power to detect between-group differences in this outcome in the context of a relatively small study (*N* = 105). This will serve as an important indicator that a larger study may lead to measurable changes in clinically relevant health outcomes such was body weight, physical activity, and DPP engagement. It is also possible that we will not detect changes in autonomous motivation. For this reason, we will conduct qualitative interviews with study participants so that we can interpret our quantitative findings in the context of individual-level participant experiences. For example, it is possible that some individuals may desire additional support (e.g., brief counseling, nutritional advice, explicit exercise goals) to augment the JOOL Health experience. Some individuals may want more tailored messaging from the JOOL Health application while others may find the daily charting to be burdensome. Understanding participants’ experiences will enable us to refine and strengthen our program. In this way, we will tailor our intervention to better meet the individual-level needs of this previously overlooked population with prediabetes who have not engaged in a formal DPP.

Importantly, we will also gain new insight into the feasibility of recruiting a target population that is presumed to be difficult to engage based on extremely low rates of uptake in offered DPP options. However, it is plausible that some individuals, although reluctant to engage in a year-long DPP, may be interested in a lower-intensity program such as this mHealth intervention. Relatively high levels of engagement in this intervention may suggest that this population is not as difficult to reach as presumed. Rather, tailored strategies and varied options to encourage weight loss and increased physical activity may be needed to help such individuals prevent type 2 diabetes. In contrast, relatively low levels of engagement may confirm our suspicion that this is truly a difficult population to engage, and we could then consider focus groups and/or interviews with key stakeholders to explore best practices for engagement.

This study has several important potential limitations. First, this is, to our knowledge, the first study to test a behavioral change program among individuals who previously declined DPP participation despite being invited to participate in either an in-person or web-based option at no out-of-pocket-cost. Accordingly, it is difficult to predict rates of recruitment or adherence among this population, as these individuals may be more reluctant to make lifestyle changes than the general population, and additional strategies may be necessary to encourage participation. We will thus collect data about the intervention’s feasibility and acceptability, and we will apply learned lessons to the subsequent larger study. Second, despite numerous mHealth applications to promote behavioral change and chronic disease management, rates of engagement with these applications over time are low [56], and use of mHealth devices may have unpredictable effects on the behaviors and outcomes they aim to modify [57, 58]. It is unknown whether study participants will engage with JOOL Health or whether use of the app will stimulate positive behavioral change among the study population. Encouragingly, JOOL Health possesses key features associated with app effectiveness (e.g., user-friendly design, real-time feedback, and individualized messages) [59], which we believe will facilitate app use and promote healthy behaviors. Lastly, our study population will only include members of the University of Michigan’s self-funded insurance plans, which may limit the generalizability of our findings. To minimize this limitation, we will recruit from a random sample of health plan beneficiaries with prediabetes, which includes over 20,000 employees, dependents, and retirees.

To reduce the public health burden of prediabetes and T2DM, novel strategies are needed to promote healthy behaviors among individuals with prediabetes. The information gathered through this study will inform subsequent work to identify clinically effective, cost-effective, and highly-scalable approaches to prevent or delay the onset of T2DM and enhance the population health impact of existing Diabetes Prevention Programs.

## Additional file

Additional file 1: SPIRIT checklist. (PDF 122 kb)

## Abbreviations

DPP: Diabetes Prevention Program
HbA1c: Hemoglobin A1c
mHealth: Mobile health
SDT: Self-Determination Theory
T2DM: Type 2 diabetes mellitus

## References

[1] Centers for Disease Control and Prevention. National Diabetes Statistics Report: Estimates and Its Burden in the United States. Available from: https://www.cdc.gov/diabetes/data/statistics/statistics-report.html. [cited 2016 May 27].

[2] 2014 Diabetes Report Card—diabetesreportcard2014.pdf. Available from: http://www.cdc.gov/diabetes/pdfs/library/diabetesreportcard2014.pdf. [cited 2016 May 27].

[3] Ackermann RT, Finch EA, Brizendine E, Zhou H, Marrero DG (2008). Translating the Diabetes Prevention Program into the community. Am J Prev Med.

[4] Vojta D, Koehler TB, Longjohn M, Lever JA, Caputo NF (2013). A coordinated national model for diabetes prevention. Am J Prev Med.

[5] Ali MK, Echouffo-Tcheugui JB, Williamson DF (2012). How effective were lifestyle interventions in real-world settings that were modeled on the Diabetes Prevention Program?. Health Aff (Millwood).

[6] Ma J, Yank V, Xiao L, Lavori PW, Wilson SR, Rosas LG (2013). Translating the Diabetes Prevention Program lifestyle intervention for weight loss into primary care: a randomized trial. JAMA Intern Med.

[7] McTigue KM, Conroy MB, Bigi L, Murphy C, McNeil M (2009). Weight loss through living well: translating an effective lifestyle intervention into clinical practice. Diabetes Educ.

[8] Whittemore R, Melkus G, Wagner J, Dziura J, Northrup V, Grey M (2009). Translating the Diabetes Prevention Program to primary care: a pilot study. Nurs Res.

[9] Lindström J, Absetz P, Hemiö K, Peltomäki P, Peltonen M (2010). Reducing the risk of type 2 diabetes with nutrition and physical activity—efficacy and implementation of lifestyle interventions in Finland. Public Health Nutr.

[10] Tuomilehto J, Lindström J, Eriksson JG, Valle TT, Hämäläinen H, Ilanne-Parikka P (2001). Prevention of type 2 diabetes mellitus by changes in lifestyle among subjects with impaired glucose tolerance. N Engl J Med.

[11] Group DPPR (2002). Reduction in the incidence of type 2 diabetes with lifestyle intervention or metformin. N Engl J Med.

[12] Dunkley AJ, Bodicoat DH, Greaves CJ, Russell C, Yates T, Davies MJ (2014). Diabetes prevention in the real world: effectiveness of pragmatic lifestyle interventions for the prevention of type 2 diabetes and of the impact of adherence to guideline recommendations: a systematic review and meta-analysis. Diabetes Care.

[13] DoIHavePrediabetes.org | Prevent Type 2 Diabetes. Available from: https://doihaveprediabetes.org/. [cited 2017 Jan 12].

[14] Prevent Diabetes STAT | Health Care Professionals. Available from: https://preventdiabetesstat.org/index.html. [cited 2017 Jan 12].

[15] Jackson SL, Long Q, Rhee MK, Olson DE, Tomolo AM, Cunningham SA (2015). Weight loss and incidence of diabetes with the Veterans Health Administration MOVE! Lifestyle Change Programme: an observational study. Lancet Diabetes Endocrinol.

[16] Lakerveld J, IJzelenber W, van Tulder MW, Hellemans IM, Rauwerda JA, van Rossum AC (2008). Motives for (not) participating in a lifestyle intervention trial. BMC Med Res Methodol.

[17] Toft UN, Kristoffersen LH, Aadahl M, von Huth SL, Pisinger C, Jorgensen T (2007). Diet and exercise intervention in a general population—mediators of participation and adherence: the Inter99 study. Eur J Pub Health.

[18] Lu S, Harris MF (2013). Prevention of diabetes and heart disease: patient perceptions on risk, risk assessment and the role of their GP in preventive care. Aust Fam Physician.

[19] Casey D, De Civita M, Dasgupta K (2010). Understanding physical activity facilitators and barriers during and following a supervised exercise programme in type 2 diabetes: a qualitative study. Monitoring and Support for Activity in Type 2 Diabetes. Diabet Med.

[20] Chen F, Su W, Becker SH, Payne M, Castro Sweet CM, Peters AL (2016). Clinical and economic impact of a digital, remotely-delivered intensive behavioral counseling program on Medicare beneficiaries at risk for diabetes and cardiovascular disease. PLoS One.

[21] Sepah SC, Jiang L, Peters AL (2014). Translating the Diabetes Prevention Program into an online social network validation against CDC standards. Diabetes Educ..

[22] Health Plans Preventing Diabetes and Improving Well-Being. AHIP. 2016. Available from: https://www.ahip.org/diabetes/. [cited 2016 May 27].

[23] Alex ADA 1701 NBS, ria, 1–800-Diabetes V 22311. National Diabetes Prevention Program Named the First Preventive Health Initiative Eligible for Medicare Coverage via CMMI Expansion. Am. Diabetes Assoc. Available from: http://www.diabetes.org/newsroom/press-releases/2016/national-dpp-named-first-preventive-health-initiative-eligible-for-medicare.html. [cited 2016 May 27].

[24] (ASPA) AS for PA. Independent experts confirm that diabetes prevention model supported by the Affordable Care Act saves money and improves health. HHS.gov. 2016. Available from: http://www.hhs.gov/about/news/2016/03/23/independent-experts-confirm-diabetes-prevention-model-supported-affordable-care-act-saves-money.html. [cited 2016 Jun 28].

[25] Kullgren JT, Knaus M, Jenkins KR, Heisler M (2016). Mixed methods study of engagement in behaviors to prevent type 2 diabetes among employees with pre-diabetes. BMJ Open Diabetes Res Care.

[26] Azar KMJ, Lesser LI, Laing BY, Stephens J, Aurora MS, Burke LE (2013). Mobile applications for weight management. Am J Prev Med.

[27] Deci EL, Ryan RM (2000). The “what” and “why” of goal pursuits: human needs and the self-determination of behavior. Psychol Inq.

[28] Koestner R, Otis N, Powers TA, Pelletier L, Gagnon H (2008). Autonomous motivation, controlled motivation, and goal progress. J Pers.

[29] Shigaki C, Kruse RL, Mehr D, Sheldon KM, Ge B, Moore C (2010). Motivation and diabetes self-management. Chronic Illn.

[30] Williams GC, Grow VM, Freedman ZR, Ryan RM, Deci EL (1996). Motivational predictors of weight loss and weight-loss maintenance. J Pers Soc Psychol.

[31] Teixeira PJ, Carraça EV, Marques MM, Rutter H, Oppert J-M, De Bourdeaudhuij I (2015). Successful behavior change in obesity interventions in adults: a systematic review of self-regulation mediators. BMC Med.

[32] Hurkmans EJ, Maes S, de Gucht V, Knittle K, Peeters AJ, Ronday HK (2010). Motivation as a determinant of physical activity in patients with rheumatoid arthritis. Arthritis Care Res.

[33] Koponen AM, Simonsen N, Suominen S. Determinants of physical activity among patients with type 2 diabetes: the role of perceived autonomy support, autonomous motivation and self-care competence. Psychol Health Med. 2017;22(3):332-44. 10.1080/13548506. 10.1080/13548506.2016.1154179 26952696

[34] Kullgren JT (2016). A mixed methods study of initial engagement in behaviors to prevent diabetes among employees with prediabetes.

[35] Teixeira PJ, Silva MN, Mata J, Palmeira AL, Markland D (2012). Motivation, self-determination, and long-term weight control. Int J Behav Nutr Phys Act.

[36] Silva MN, Markland D, EV CA, Vieira PN, Coutinho SR, Minderico CS (2011). Exercise autonomous motivation predicts 3-yr weight loss in women. Med Sci Sports Exerc.

[37] What Is JOOL? – JOOL Health. Available from: http://www.joolhealth.com/what-is-jool/. [cited 2017 Jun 14].

[38] Asimakopoulos S, Asimakopoulos G, Spillers F (2017). Motivation and user engagement in fitness tracking: heuristics for mobile healthcare wearables. Informatics.

[39] Tashakkori A, Teddlie C. Handbook of Mixed Methods in Social & Behavioral Research. SAGE. 2003.

[40] Ivankova NV, Creswell JW, Stick SL (2006). Using mixed-methods sequential explanatory design: from theory to practice. Field Methods..

[41] REDCap. Available from: https://www.project-redcap.org/. [cited 2017 Jun 19].

[42] Levesque CS, Williams GC, Elliot D, Pickering MA, Bodenhamer B, Finley PJ (2006). Validating the theoretical structure of the treatment self-regulation questionnaire (TSRQ) across three different health behaviors. Health Educ Res.

[43] Thomas J, Harden A (2008). Methods for the thematic synthesis of qualitative research in systematic reviews. BMC Med Res Methodol.

[44] Francis JJ, Johnston M, Robertson C, Glidewell L, Entwistle V, Eccles MP (2010). What is an adequate sample size? Operationalising data saturation for theory-based interview studies. Psychol Health.

[45] Scheier MF, Wrosch C, Baum A, Cohen S, Martire LM, Matthews KA (2006). The life engagement test: assessing purpose in life. J Behav Med.

[46] Ryff CD, Keyes CLM (1995). The structure of psychological well-being revisited. J Pers Soc Psychol.

[47] 1998_WilliamsFreedmanDeci.pdf. Available from: http://selfdeterminationtheory.org/SDT/documents/1998_WilliamsFreedmanDeci.pdf. [cited 2016 Jun 20].

[48] Sarason IG, Sarason BR, Shearin EN, Pierce GR (1987). A brief measure of social support: practical and theoretical implications. J Soc Pers Relatsh.

[49] de Lauzon B, Romon M, Deschamps V, Lafay L, Borys J-M, Karlsson J (2004). The three-factor eating questionnaire-R18 is able to distinguish among different eating patterns in a general population. J Nutr.

[50] Craig CL, Marshall AL, Sjöström M, Bauman AE, Booth ML, Ainsworth BE (2003). International physical activity questionnaire: 12-country reliability and validity. Med Sci Sports Exerc.

[51] Roberts NJ, Kidd L, Dougall N, Patel IS, McNarry S, Nixon C (2016). Measuring patient activation: the utility of the patient activation measure within a UK context—results from four exemplar studies and potential future applications. Patient Educ Couns.

[52] REDCap. Available from: https://www.project-redcap.org/. [cited 2017 Sep 27].

[53] Hsieh H-F, Shannon SE (2005). Three approaches to qualitative content analysis. Qual Health Res.

[54] Chinn DJ, White M, Howel D, Harland JOE, Drinkwater CK (2006). Factors associated with non-participation in a physical activity promotion trial. Public Health.

[55] Groeneveld IF, Proper KI, van der Beek AJ, Hildebrandt VH, van Mechelen W (2009). Factors associated with non-participation and drop-out in a lifestyle intervention for workers with an elevated risk of cardiovascular disease. Int J Behav Nutr Phys Act.

[56] Ernsting C, Dombrowski SU, Oedekoven M, O’Sullivan JL, Kanzler M, Kuhlmey A (2017). Using smartphones and health apps to change and manage health behaviors: a population-based survey. J Med Internet Res.

[57] Jakicic JM, Davis KK, Rogers RJ, King WC, Marcus MD, Helsel D (2016). Effect of wearable technology combined with a lifestyle intervention on long-term weight loss: the IDEA randomized clinical trial. JAMA.

[58] Lyons RA, Rodgers SE, Thomas S, Bailey R, Brunt H, Thayer D (2016). Effects of an air pollution personal alert system on health service usage in a high-risk general population: a quasi-experimental study using linked data. J Epidemiol Community Health.

[59] Zhao J, Freeman B, Li M (2016). Can mobile phone apps influence people’s health behavior change?. An Evidence Review J Med Internet Res.

